# Bufadienolides from* Venenum Bufonis* Inhibit mTOR-Mediated Cyclin D1 and Retinoblastoma Protein Leading to Arrest of Cell Cycle in Cancer Cells

**DOI:** 10.1155/2018/3247402

**Published:** 2018-07-11

**Authors:** Huamei Chang, Juan Li, Yajun Cao, Tiantian Liu, Suofang Shi, Wenxing Chen

**Affiliations:** ^1^Jiangsu Pujin Pharmaceutical Co., Ltd., Nanjing, China; ^2^School of Pharmacy, Hunan University of Chinese Medicine, Changsha, China; ^3^Jiangsu Key Laboratory for Pharmacology and Safety Evaluation of Chinese Materia Medica, China; ^4^School of Pharmacy, Nanjing University of Chinese Medicine, Nanjing, China; ^5^Department of Respiration, The First Clinical Medical School, Nanjing University of Chinese Medicine, Nanjing, China

## Abstract

**Objective:**

Bufadienolides, the main components in* Venenum Bufonis* secreted from toads, have been proved to be with significant anticancer activity aside from the positive inotropic action as cardenolides. Here an underlying anticancer mechanism was further elucidated for an injection made from* Venenum Bufonis* containing nine bufadienolides.

**Methods:**

One solution reagent and cell cycle analyses were for determining effect of bufadienolides on cancer cells. Western blotting was used for protein expression.

**Results:**

Bufadienolides inhibit cell proliferation and arrest cells in G1 phase. Bufadienolides also inhibit the mammalian target of rapamycin (mTOR) signaling pathway, which is evidenced by the data that bufadienolides inhibit type I insulin-like growth factor- (IGF-1-) activated phosphorylation of mTOR by a concentration- and time-dependent way, as well as phosphorylation of p70 S6 kinase 1 (S6K1) and eukaryotic initiation factor 4E (eIF4E) binding protein 1 (4E-BP1). Subsequent results indicated that cyclin D1 expression and phosphorylation of retinoblastoma protein (Rb)—two characterized regulators in cell cycle of G1—are also inhibited and the process is dependent on mTOR pathway.

**Conclusion:**

Bufadienolides inhibit proliferation partially due to arresting cell cycle in G1 phase, which is mediated by inhibiting mTOR-cyclin D1/Rb signal pathway.

## 1. Introduction

The toad is an amphibious animal and its white dried secretion also called Chansu in Chinese was considered as a traditional medicine for mainly curing infectious disease thousands years ago in China [1]. Nowadays, Chansu was demonstrated to possess many activities and has been used for cardiac disease [2, 3] and inflammation. Particularly, much concern has been focused on its anticancer activity. Lots of studies have proved that Chansu, specially its main components such as bufalin, cinobufagin, and resibufogenin, shows significant inhibition on a series of cancer cells [4]. Bufalin could induce apoptosis by inhibiting Bcl2 and activating Bax expression via the mitochondria dependent pathway in human tongue cancer cells [5, 6]. Bufalin suppresses pancreatic cancer by causing cell cycle arrested in S phase through targeting the c-Myc [7]. Furthermore, bufalin also shows an inhibition on metastasis in human lung cancer cells dependent on blocking MMPs, MAPKs, and NF-*κ*b signaling pathway [8]. Cinobufagin indicates antiproliferative effect on U266 multiple myeloma cells through regulating ROS-activated MAPKs pathway [9]. Cinobufagin could induce apoptosis in osteosarcoma cells by inactivation of notch [10] and cause cell death in human osteosarcoma U2OS cells via the ROS-mediated autophagy and JNK/p38 pathway [11]. Cinobufagin also inhibits tumor growth through inducing AKT/mTOR-mediated intrinsic apoptosis in human non-small cell lung cancer cells [12]. In addition, resibufogenin could degrade cyclin D1 to induce G1-phase arrested in human malignant cancer cells [13] and have a prevention of oxidative stress in a rat model of human preeclampsia [14]. The above-mentioned studies suggested that most of individual component in Chansu has anticancer activity.

**Figure 1:**
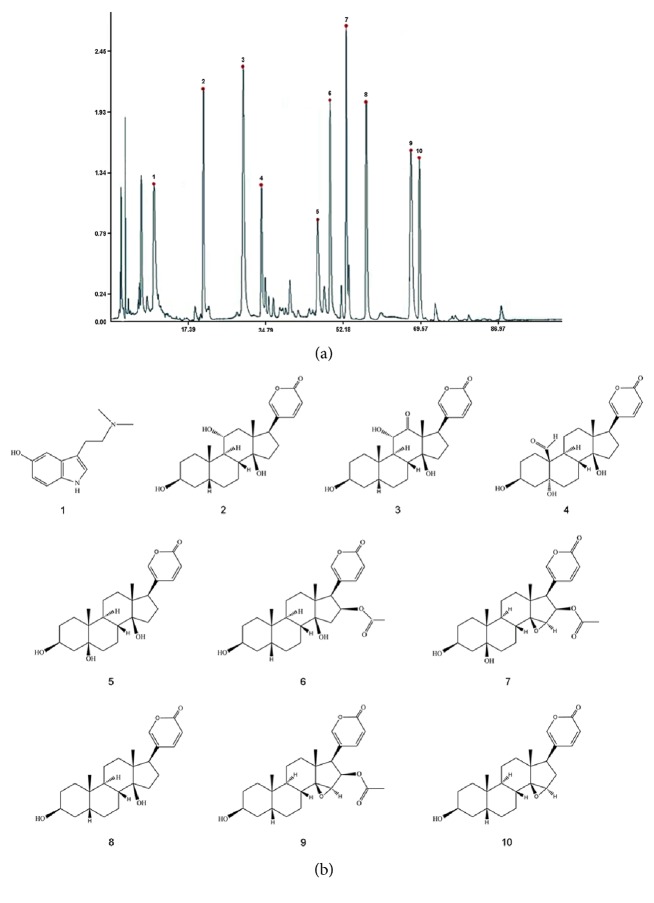
**The Chansu injection information. **(a) The fingerprint graph of Chansu injection. (b) The structure of identified compounds including nine bufadienolides.

Chansu injection, a CFDA-approved injection, was made from* Venenum Bufonis* and used for clinical inflammation and fever in China. And it was composed of at least nine bufadienolides which have been identified by HPLC (Figures 1(a)-1(b), Table 1). However, more previous research was focused on the single bufadienolide such as bufalin, cinobufagin, and resibufogenin rather than on the whole injection. Most of single bufadienolide has antitumor action, and Chansu, a mixture of many bufadienolides, also has been proved to be with same activity [15–17]. However, its anticancer mechanism has been rarely concerned. So it is worthy to further elucidate its underlying mechanism for guiding the clinical development and use. In this article, we found that bufadienolides could inhibit proliferation of cancer cells via arresting cancer cell cycle in G1 phase, which resulted from the decreased expression of cyclin D1 and Rb phosphorylation. Subsequently, mTOR and mTOR-mediated S6K1 and 4E-BP1 signal pathway were inhibited by bufadienolides during this course.

## 2. Materials and Methods

### 2.1. Materials

Dulbecco's Modified Eagle Medium (DMEM) and fetal bovine serum (FBS) were purchased, respectively, from Mediatech (Herndon, VA, USA) and Gibco (Logan, UT, USA). Trypsin was from Invitrogen (Grand Island, NY, USA). Type I insulin-like growth factor (IGF-I) (PeproTech, NJ, USA) was rehydrated in 0.1 M acetic acid for preparing a stock solution (10 ng/ml), aliquoted, and stored at -80°C. The following antibodies were used: mTOR, phospho-mTOR (Ser2448), S6K1, phospho-p70 S6K1 (Thr389), 4E-BP1, Cyclin A, CDK2, Cyclin B1, CDK4, Cyclin D1, Cyclin E, p-Rb, and *β*-tubulin (Cell Signaling, Beverly, MA, USA).

Chansu injection, provided by Jiangsu Pujin pharmaceutical Co., Ltd., contains major compounds including nine bufadienolides which accounts for more than 90%. And per 1ml Chansu injection contains 96*μ*g bufadienolides. So in this paper, the Chansu injection was represented by the bufadienolides.

### 2.2. Cell Lines and Cultures

Cell lines of human breast cancer (MCF-7) and prostate cancer (DU145) cells were cultured in antibiotic-free DMEM containing 10% FBS. Both cell lines were incubated in a 37°C incubator with 5% CO_2_. For different experiments, the different cell lines were disposed with different ways.

### 2.3. Cell Proliferation

Cells by a density of 1 × 10^4^ cells/well were seeded in 96-well plates for 24h before incubation. Then various concentrations of bufadienolides (0-20 *μ*g/ml) were added for 48h treatment with six replicates of each concentration. After incubation, 20 *μ*L of one solution reagent (Promega) was added into the plate which was continuously incubated for 4 h. Cell viability was measured and calculated by the optical density at 490 nm from a BioTek reader (BioTek, USA). The inhibition rate (%) = (A_control_-A_sample_)/A_control_*∗*100%. Cells (1 × 10^5^ cells/well) grown in 6-well plates and treated as above were prepared for cell morphological images taken by Zeiss inverted phase-contrast microscope.

### 2.4. Cell Cycle Analysis

MCF-7 cells grown in 100 mm dishes (1× 10^6^ cells/dish) with 10ml RPMI-1640 containing 10% FBS were grown overnight in a humidified 37°C incubator with 5% CO_2_. Cells were then cultured with various concentrations of bufadienolides (0-20 *μ*g/ml) for 24 h. Finally, cell cycle analysis was executed on the treated cells by the Cellular DNA Flow Cytometric Analysis Kit (BD Corp., Franklin Lakes, NJ, USA).

### 2.5. IGF-I Activated mTOR Signal Pathway

Cells dispersed in DMEM with 10% FBS and seeded in 6-well plates by a density of 1×10^6^ cells/well were cultured in serum-free DMEM for 24-h starvation at 37°C and 5% CO_2_ incubator. Then cells were treated with 0-10 *μ*g/ml bufadienolides for 2 h, followed with one hour stimulation/nonstimulation by IGF-I (10 ng/ml). The cells were harvested for western blotting analysis using the indicated antibodies in final.

### 2.6. Western Blot Analysis

Monolayer cells were lysed in solution of RIPA buffer. After 15-s sonication, lysates were coldly centrifuged at 13,000 rpm for 2 min. Sample protein concentration was tested by bicinchoninic acid kits and bovine serum albumin acted as standard (Pierce, IL, USA). Equivalent weight of protein (approximately 40*μ*g) was separated on 6-12% SDS-polyacrylamide gel and then transferred to polyvinylidene difluoride membranes (Millipore, MA, USA). Membranes with target protein were immersed in 5% nonfat milk solution supplemented with 0.05% Tween-20 for covering nonspecific protein. Finally, the membranes were incubated in solution with primary antibodies overnight and then with corresponding secondary antibodies conjugated with horseradish peroxidase. Immunoblotting bands were emerged by enhanced chemiluminescence reagent (Perkin-Elmer, MA, USA).

### 2.7. Statistical Analysis

All values were represented as mean±sd. The data were analyzed by one-way analysis of variance (ANOVA) for significant difference. A level of p<0.05 was considered statistically significant.

## 3. Results

### 3.1. Bufadienolides Inhibit Cancer Cell Proliferation

**Figure 2:**
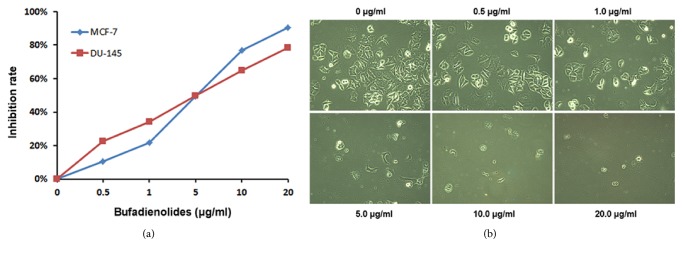
**Bufadienolides inhibit cancer cell proliferation. **Bufadienolides inhibit cancer cell proliferation in a dose-dependent way. (a) MCF-7 and DU-145 cells, grown in 96-well plates with 10% FBS-DMEM medium, were treated with bufadienolides (0-20 *μ*g/ml) for 48 h. Cell proliferation was assessed using one solution reagent (Promega). Absorbance at 490 nm was determined by a Biotek Multilabel Counter. (b) The representative photograph was taken by ZEISS microscope (× 200).

We systematically screened the cytotoxicity of bufadienolides on several cancer cells and found that bufadienolides have the apparent inhibition on many human cancer cells. As indicated in Figure 2(a), the inhibition rate for two cancer cells treated with bufadienolides (0-20 *μ*g/ml) for 48 h indicates a dose-dependent increase, suggesting that cells viability is inhibited.  Figure 2(b) shows the representative photos in MCF-7 cells. The IC50 for MCF-7 cells is about 4.57 *μ*g/ml and for DU145 cells approximately 4.69 *μ*g/ml.

### 3.2. Bufadienolides Arrest Cell Cycle in G1 Phase

**Figure 3:**
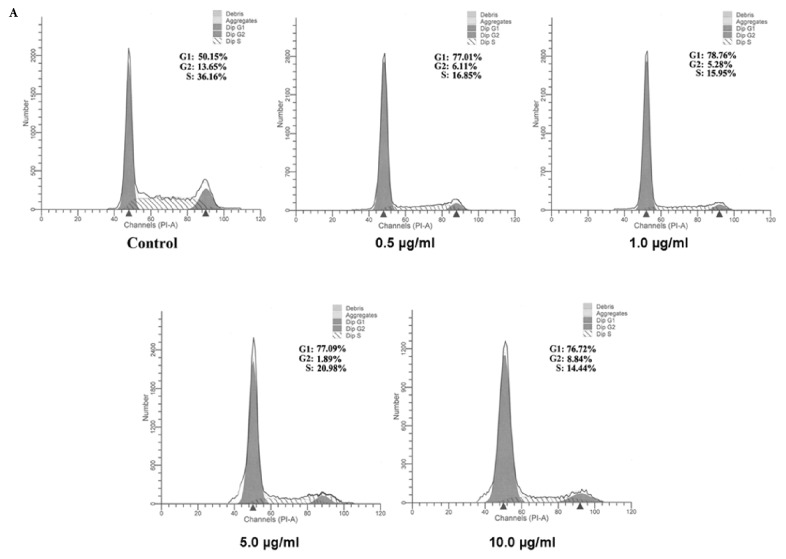
**Bufadienolides arrest cell cycle in G1 phase. **MCF-7 cells, grown in 10% FBS-RPMI medium in 6-well plates (1×10^5^ cells/well), were treated with bufadienolides (0-10*μ*g/ml) for 24 h, followed by cell cycle analysis.

It is well known that cell proliferation need experience four phases including G1, S, G2, and M to complete a cleavage. Each phase is very important for cell progression. If any phase in a cycle is interfered by external factors, the cell proliferation would be terminated and the cells go to death finally. To understand whether the bufadienolides block the cell cycle and at which phase is aimed if blocking, we checked the number of cells stained by propidium iodide in each phase of cell cycle using the Flow Cytometer. The results showed that the rate of MCF-7 cells in G1, S, and G2 phase, respectively, is 50.15%, 36.16%, and 13.65% in control group (Figure 3). However, in treatment group with bufadienolides, it in G1 phase runs a significant increase from 0.5 *μ*g/ml to 10 *μ*g/ml and a significant decrease in S phase, suggesting that the cell cycle from G1 to S was stopped in a large scale. Bufadienolides block the cell cycle in G1 phase.

### 3.3. Bufadienolides Inhibit Cyclin D1 and Rb Phosphorylation

**Figure 4:**
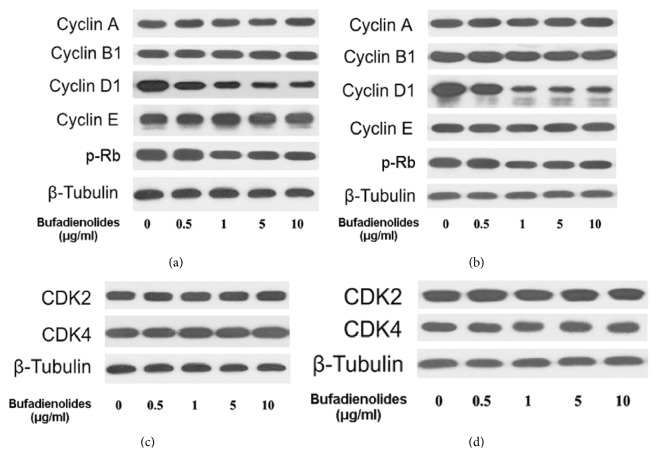
**Bufadienolides inhibit expression of cyclin D1 and phosphorylation of Rb**. (a, c) MCF-7 cells were treated with different concentration of bufadienolides (0-10 *μ*g/ml) for 24 h, then cellular lysates were subjected to western blotting analysis for detecting the indicated protein molecules. (b, d) The same experiment was repeated in DU145 cells.

Many Cyclins and CDKs, as well as CDK inhibitors, are involved in regulating cell cycle. Since the bufadienolides could induce cell cycle stopping in G1 phase, we further wanted to know how these factors of cell cycle change. So we collected the cell lysates from cancer cells treated with bufadienolides for western blotting. The western blot analysis indicated that cyclin D1 expression is inhibited by a dose-dependent way and Rb goes decreasingly in contrast to the increasing dose of bufadienolides in MCF-7 cells (Figure 4(a)). At 1 *μ*g/ml, bufadienolides began to show obvious inhibition of cyclin D1 and phosphorylation of Rb and are significant at more than 5*μ*g/ml compared with control. Other cyclin proteins including cyclin A, cyclin B1, and cyclin E show no significant change, which just matches the conclusion from analysis of flow cytometer (Figure 4(a)). In addition, similar results were also got in DU145 cells starting from dose of 1*μ*g/ml (Figure 4(b)). The expression of CDK2 and CDK4 which regulate the cyclins in MCF-7 and DU145 cells has no variation by the treatment of bufadienolides (Figures 4(c) and 4(d)).

### 3.4. Bufadienolides Inhibit IGF-1 Activated mTOR Pathway

**Figure 5:**
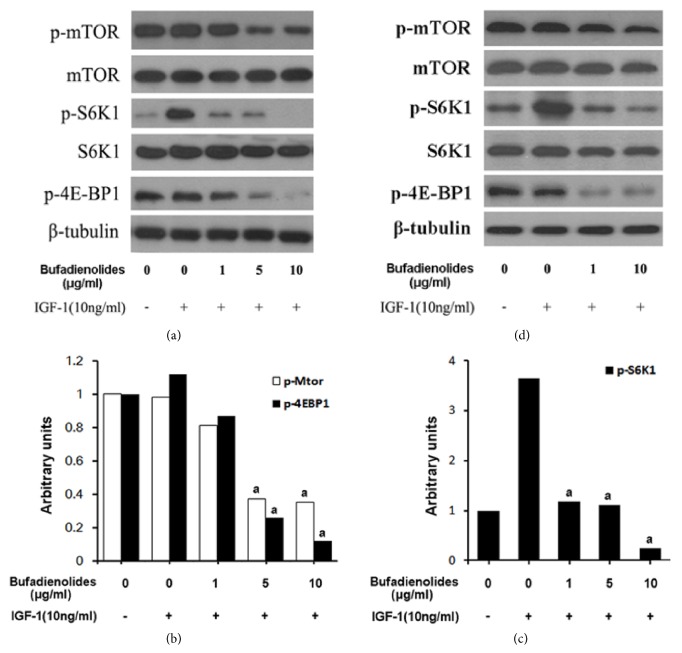
**Bufadienolides inhibit mTOR pathway by a concentration-dependent manner.** Serum-free cultured MCF-7 (a) and DU145 (d) cells were pretreated with bufadienolides (0-10 *μ*g/ml) for 2 h, then stimulated with IGF-1 (10 ng/ml) for 1 h, and finally subjected to western blotting analysis with indicated antibodies. (b, c) Densitometry analysis of p-mTOR, p-S6K1, and p-4E-BP1 bands in MCF-7 cells was normalized to *β*-tubulin using ImageJ, expressed as arbitrary units versus IGF-1(10 ng/ml), a: p<0.01.

mTOR, as a therapeutic target against cancer, plays a pivotal role in cellular proliferation, growth, and survival. It is also one of the important upstream regulators controlling the cell cycle progression. Is it possible for bufadienolides to inhibit mTOR pathway? For confirming the hypothesis, we selected IGF-1 as a mediated factor which activates PI3K-mTOR-S6K1/4E-BP1 pathway and began to determine the effect of bufadienolides on the mTOR signal pathway in MCF-7 cells. As the central molecule of mTOR pathway, it is the key to judge whether the compound has an effect on phosphorylation of mTOR. The mTOR has two phosphorylated sites, Ser2448 phosphorylated by AKT and autophosphorylated Ser2481. Our data indicates that bufadienolides inhibited phosphorylation of Ser2448 but not Ser2481 (Figure 5(a)). Furthermore, phosphorylation of S6K1, one of the best-characterized downstream proteins of mTOR, was inhibited by bufadienolides in a dose-dependent manner from 1 *μ*g/ml to 10 *μ*g/ml in MCF-7 cells (Figure 5(a)), as well as the phosphorylation of 4E-BP1. Densitometry analysis also demonstrates the above results (Figures 5(b) and 5(c)). Altogether, bufadienolides potently inhibit p-mTOR and mTOR-mediated p-S6K1 and p-4E-BP1.

### 3.5. Inhibition of mTOR Involved in Bufadienolides Arresting Cell Cycle in G1

**Figure 6:**
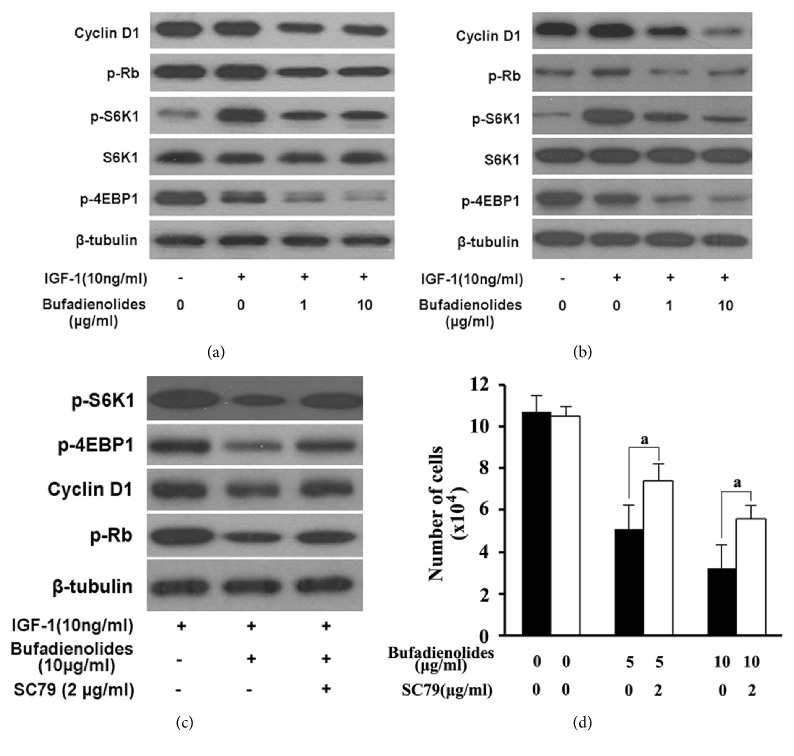
**Bufadienolides inhibition of cyclin D1 is mediated by mTOR**. Serum-free cultured MCF-7 (a) and DU145 (b) cells were pretreated with bufadienolides (0-10 *μ*g/ml) for 2 h, then stimulated with IGF-1 (10 ng/ml) for 1 h, and finally subjected to western blotting analysis. (c) MCF-7 cells were pretreated with or without SC79 (2*μ*g/ml) for 1h and then treated with bufadienolides (10 *μ*g/ml) for 2 h, followed by IGF-1 (10 ng/ml) stimulation for 1 h, and finally subjected to western blotting analysis. (d) MCF-7 cells were pretreated with or without SC79 (2*μ*g/ml) for 1h and then treated with bufadienolides (10 *μ*g/ml) for 24 h, followed by cell counting analysis. a indicates p<0.01.

Cyclin D1 could be activated through SP1, c-Jun transfactors mediating MAPK cascade or through AKT/NF-*κ*B pathway [18, 19]. AKT may prevent from degrading cyclin D1 via inhibition of GSK-3*β*-dependent pathway [20]. Additionally, cyclin D1 also was regulated through mTOR-S6K1/4E-BP1 pathway [21]. Although we have found above that bufadienolides inhibited the mTOR-S6K1/4E-BP1 pathway, it is unconfirmed that bufadienolides resulting in cancer cell cycle arrested in G1 phase were completely or partially mediated by suppression of mTOR pathway. Cyclin D1 is induced by growth factors including EGF, IGF-I, IGF-II, and amino acids [22]. Generally, activation of IGF-IR by IGF-I could activate PI3K and MAPK pathway, following the subsequent activation of mTOR pathway [23]. Therefore, we examined the mTOR and cyclin D1 simultaneously in an IGF-I-stimulated condition, as well as their downstream protein molecules. As shown in Figures 6(a) and 6(b), treated with different dose of bufadienolides, p-S6K1 and p-4E-BP1 were inhibited in 2 h following the similar tendency on cyclin D1 and p-Rb in MCF-7 and DU145 cells. For further demonstration of their relationship, SC79, an AKT activator for activating mTOR-cyclin D1 pathway [24], was used to pretreat the cells before bufadienolides treatment. The data of Figure 6(c) indicated that 10 *μ*g/ml bufadienolides could inhibit the expression of p-S6K1, p-4EBP1, cyclin D1, and p-Rb, while the expression inhibition could be partially reversed by 2 *μ*g/ml SC79, and the viable cells number of groups of bufadienolides plus SC79 also are significantly more than the bufadienolides group (Figure 6(d)). These concluded that bufadienolides inhibition of mTOR signal pathway was associated with its suppression of cyclin D1 and p-Rb which induces cell cycle arrested in G1 phase.

## 4. Discussion

The eukaryotic cell cycle consists of four discrete phases: G1 (gap phase 1), S (DNA synthesis phase), G2 (gap phase 2), and M (mitosis) [25]. Progression of cell cycle is controlled by many protein molecules including cyclins, cyclin-dependent kinases (CDKs), and CDK inhibitors [26, 27]. In mammalian cells, many cyclins are involved in controlling cell cycle transitions. Cyclin D1 controls progression of G1 phase, while cyclin E promotes G1 transition to S phase. Furthermore, cyclin A and cyclin B, respectively, were responsible for S-phase progression and for entry into M phase. Among them, cyclin D1 is regarded as a central protein for cell cycle progression in G1 [28], playing a crucial role in controlling cell cycle from G1 to S phase. Overexpression of cyclin D1 was tightly related to many types of cancer such as breast cancer, lung cancer, colon cancer, and prostate cancer [29–31]. The cell cycle begins from G1 phase with increased expression of the cyclin D1 [32]. The cyclin D1 binding to CDK4 and forming the cyclin/CDK complexes result in phosphorylation and activation of the CDKs [33]. Then the retinoblastoma (Rb) protein, product of the retinoblastoma gene (Rb-I) and targeting the E2F transcription factors, was phosphorylated by the activated CDKs [33]. And Rb also functions as a negative controller of the cell cycle in its unphosphorylated form during G1 [34]. Rb phosphorylated by CDKs in G1 induces release of some transcription factors that are necessary for regulating the genes controlling the transition from G1 to S and subsequent DNA synthesis [35, 36].

In experiments, we found that bufadienolides block the progression of cell cycle in G1 phase, followed by inhibition of cyclin D1 and phosphorylation of Rb but not any other molecules. Noticeably, decreased cyclin D1 leads to reduction of cyclin D1-CDK4 complexes and its part inactivation. Subsequent phosphorylation of pRb also was reduced, which is due to the cell cycle arrested in G1. Significant changes were not discovered in cyclin A, cyclin B1, cyclin E, and so on, which is just another convictive evidence for bufadienolides to target the G1 phase. Furthermore, bufalin, one of the bufadienolides, has been found to induce cell cycle arrested in G0/G1 phase by inhibition of cyclin D1 and related CDKs [37], and resibufogenin accelerates proteasomal degradation of cyclin D1 to arrest G1 phase [13], convincing that the chemicals of Chansu injection inducing G1-cell cycle arrest must be bufadienolides.

Many signal pathways are involved in regulating cell cycle through mediating the cyclin D1. NF-*κ*B, one of the important pathways regulating cell cycle, is necessary to induce cyclin D1 expression and hyperphosphorylation of Rb and promote progression of G1 to S phase [38]. Mitogen-activated protein kinase (MAPK) cascades via c-fos, c-jun are believed to increase cyclin D1 expression that occurred simultaneously with Rb phosphorylation, which promotes cell cycle progression [39]. However, the mTOR pathway is the most important way to control the cell cycle [40], playing a critical role in cell proliferation, growth, survival, and angiogenesis [40]. Activated mTOR promotes the phosphorylation of 4E-BP1 and the ribosomal protein p70 S6K. 4EBP1 binding tightly to eIF4E (Eukaryotic Translation Initiation Factor-4E) in its unphosphorylated state inhibits eIF4E activity in the initiation of protein synthesis. Phosphorylated 4E-BP1 by mTOR reduces its affinity to eIF4E, activating the proliferative response and the transition from G1 to S phase in cell cycle by mediating a cascade effect [41, 42].

Bufalin increasing the sensitivity to mTOR-induced autophagy has been previously proved in human hepatocellular carcinoma cells [43], and arenobufagin induces apoptosis and autophagy via inhibiting PI3K/Akt/mTOR [44]. Our experimental data indicated the similar results: bufadienolides inhibit mTOR pathway by simultaneous suppression of phosphorylation of mTOR(2448), S6K1, and 4E-BP1. Meanwhile, cyclin D1 and Rb are proved to change toward the direction of retarding the cell cycle. And both inhibitions could be reversed in part by AKT activator. Accordingly, it occurs to us that the primary mechanism of bufadienolides arresting the cell cycle in G1 is attributed to its inhibition of mTOR-cyclin D1/Rb pathway.

## Figures and Tables

**Table 1:** The main compounds in Chansu Injection.

No.	Name	Molecular formula	Molecular weight (g/mol)
1	Bufotenin	C12H16N2O	204.3
2	Gamabufotalin	C24H34O5	402.5
3	Arenobufagin	C24H32O6	416.5
4	Hellebrigenin	C24H32O6	416.5
5	Telocinobufagin	C24H34O5	402.5
6	Bufotalin	C26H36O6	444.6
7	Cinobufotalin	C26H34O7	458.5
8	Bufalin	C24H34O4	386.5
9	Cinobufagin	C26H34O6	442.5
10	Resibufogenin	C24H32O4	384.5

## Data Availability

All data generated or analysed during this study are included in this article.

## Abbreviations

DMEM: Dulbecco's Modified Eagle Medium
FBS: Fetal bovine serum
IGF-I: Insulin-like growth factor type I
PBS: Phosphate-buffered saline
mTOR: Mammalian target of rapamycin
4E-BP1: Eukaryotic initiation factor 4E (eIF4E) binding protein 1
NF-*κ*B: Nuclear factor kappa B
ODC: Ornithine decarboxylase
PKC: Protein kinase C
S6K1: p70 S6 kinase 1
Rb: Retinoblastoma protein
MAPK: Mitogen-activated protein kinase
CDK: Cyclin-dependent kinase
PI3K: Phosphatidylinositol 3-kinase
GSK3: Glycogen synthase kinase 3
GFP: Green fluorescence protein
CFDA: China Food and Drug Administration.
